# Small Molecule Control of Virulence Gene Expression in *Francisella tularensis*

**DOI:** 10.1371/journal.ppat.1000641

**Published:** 2009-10-30

**Authors:** James C. Charity, LeeAnn T. Blalock, Michelle M. Costante-Hamm, Dennis L. Kasper, Simon L. Dove

**Affiliations:** 1 Division of Infectious Diseases, Children's Hospital, Harvard Medical School, Boston, Massachusetts, United States of America; 2 Department of Microbiology and Molecular Genetics, Harvard Medical School, Boston, Massachusetts, United States of America; 3 Channing Laboratory, Department of Medicine, Brigham and Women's Hospital, Harvard Medical School, Boston, Massachusetts, United States of America; Dartmouth Medical School, United States of America

## Abstract

In *Francisella tularensis*, the SspA protein family members MglA and SspA form a complex that associates with RNA polymerase (RNAP) to positively control the expression of virulence genes critical for the intramacrophage growth and survival of the organism. Although the association of the MglA-SspA complex with RNAP is evidently central to its role in controlling gene expression, the molecular details of how MglA and SspA exert their effects are not known. Here we show that in the live vaccine strain of *F. tularensis* (LVS), the MglA-SspA complex works in concert with a putative DNA-binding protein we have called PigR, together with the alarmone guanosine tetraphosphate (ppGpp), to regulate the expression of target genes. In particular, we present evidence that MglA, SspA, PigR and ppGpp regulate expression of the same set of genes, and show that *mglA*, *sspA*, *pigR* and ppGpp null mutants exhibit similar intramacrophage growth defects and are strongly attenuated for virulence in mice. We show further that PigR interacts directly with the MglA-SspA complex, suggesting that the central role of the MglA and SspA proteins in the control of virulence gene expression is to serve as a target for a transcription activator. Finally, we present evidence that ppGpp exerts its effects by promoting the interaction between PigR and the RNAP-associated MglA-SspA complex. Through its responsiveness to ppGpp, the contact between PigR and the MglA-SspA complex allows the integration of nutritional cues into the regulatory network governing virulence gene expression.

## Author Summary

Guanosine tetraphosphate (ppGpp) is a small molecule that is produced by many different bacteria in response to nutrient limitation. Although ppGpp has been shown to play an important role in controlling the expression of virulence genes in several pathogenic bacteria, few studies have addressed how this occurs. Here we show that in the intracellular pathogen *F. tularensis*, ppGpp plays a critical role in controlling the expression of genes required for intracellular replication and virulence, and we uncover the molecular basis for its effect. In particular, we show that ppGpp works in concert with three other essential regulators of virulence gene expression in *F. tularensis*—a putative DNA-binding protein that we have called PigR and the SspA protein family members MglA and SspA. Our study provides evidence that ppGpp functions to promote the interaction between PigR and a component of *F. tularensis* RNA polymerase (RNAP) comprising the MglA and SspA proteins. By influencing the interaction between PigR and the RNAP-associated MglA-SspA complex, ppGpp serves to tie the nutritional status of the cell to the expression of genes that are essential for survival in the host.

## Introduction

*Francisella tularensis*, the aetiological agent of tularemia, is one of the most infectious bacterial pathogens currently known and a potential bioweapon. Although relatively little is known about the molecular mechanisms underlying *F. tularensis* pathogenesis [1], it is clear that genes present on the *Francisella* pathogenicity island (FPI) are essential for the intramacrophage growth and virulence of the organism [2]–[9]. These genes are thought to encode a novel protein secretion system related to the recently identified type VI secretion system [8], [10]–[13].

Prominent amongst those regulators of virulence gene expression in *F. tularensis* [14]–[17] are the related global regulators MglA and SspA [4], [18]–[20]. MglA and SspA work in concert with one another to positively regulate the expression of all of the genes on the FPI, and many genes outside of the FPI (∼100 genes in total) [4],[19],[20]. Moreover, recent proteomic studies have revealed that MglA influences the abundance of proteins involved in responding to stress [21]. Although MglA and SspA control the expression of many genes implicated in virulence [4],[19],[20], they also control the expression of many others whose roles in virulence are not currently known.

MglA and SspA are members of the stringent starvation protein A family [18],[22],[23] and interact directly with one another to form a complex [20]. The MglA-SspA complex in turn associates with RNA polymerase (RNAP) to positively control virulence gene expression in *F. tularensis* [20]. Although the association of the MglA-SspA complex with RNAP is evidently central to its role in controlling gene expression, the molecular details of how MglA and SspA exert their effects on transcription are not known. Members of the SspA protein family also appear to play important roles in the control of virulence gene expression in other pathogenic bacteria by unknown mechanisms [24]–[27]. Although a putative DNA-binding protein called FevR was recently shown to regulate the same set of genes that is regulated by MglA and SspA in *F. novicida*, the mechanism by which FevR influences the expression of target genes was not determined [15].

Guanosine tetraphosphate, otherwise known as ppGpp (or magic spot), is a small molecule that in some Gram-negative bacteria binds directly to RNAP and inhibits transcription initiating from certain particularly strong promoters, such as the ribosomal RNA (rRNA) promoters (reviewed in [28]–[30]). ppGpp can also directly activate transcription from other promoters by influencing the association of RNAP with the promoter [31]. In *E. coli*, the effects of ppGpp on transcription (both positive and negative) are potentiated by DksA, a small protein that binds the so-called secondary channel of RNA polymerase (the channel through which nucleotides are thought to gain access to the RNAP active center) [31]–[34].

ppGpp has been referred to as an alarmone because its intracellular concentration can change in response to a variety of stress signals, with a concomitant change in the gene expression profile of the cell (reviewed in [28]–[30]). In *E. coli*, the intracellular concentration of ppGpp is determined by the products of the *relA* and *spoT* genes. RelA is a ppGpp synthetase, which makes ppGpp in response to amino acid starvation. RelA thus mediates the so-called stringent response whereby amino acid starvation results in a reduction in rRNA expression, and a concomitant reduction in protein synthesis (reviewed in [28]–[30]). SpoT is a bifunctional protein that is able to both synthesize and degrade ppGpp. SpoT is thought to respond to conditions of carbon, fatty acid, and iron limitation [35],[36]. ppGpp plays important roles in controlling virulence gene expression in a wide variety of pathogenic bacteria, including *Legionella pneumophila* [37], *Mycobacterium tuberculosis* [38], *Salmonella* [39],[40], *Campylobacter jejuni* [41], *Pseudomonas aeruginosa* [42], *Brucella melitensis* [43], enterohaemorrhagic *E. coli* [44], *Helicobacter pylori* [45], and *Enterococcus faecalis* [46]. However, few studies have addressed how ppGpp mediates its effects on transcription in these pathogens.

Here we show that in *F. tularensis*, ppGpp and a putative DNA-binding protein we have called PigR (the ortholog of FevR from *F. novicida* [15]) work together with the MglA-SspA complex to control a common set of genes. Furthermore, we uncover the molecular bases for the coordinate activity of these transcription factors. In particular, we show that PigR interacts directly with the MglA-SspA complex and present evidence that ppGpp promotes this interaction. Our findings point to the interaction between PigR and the MglA-SspA complex being a critical regulatory checkpoint whose responsiveness to ppGpp allows the integration of nutritional cues into the transcriptional network governing *Francisella* virulence gene expression.

## Results

### The MglA-SspA complex and ppGpp positively regulate the same set of genes in *F. tularensis*

**Figure 1:**
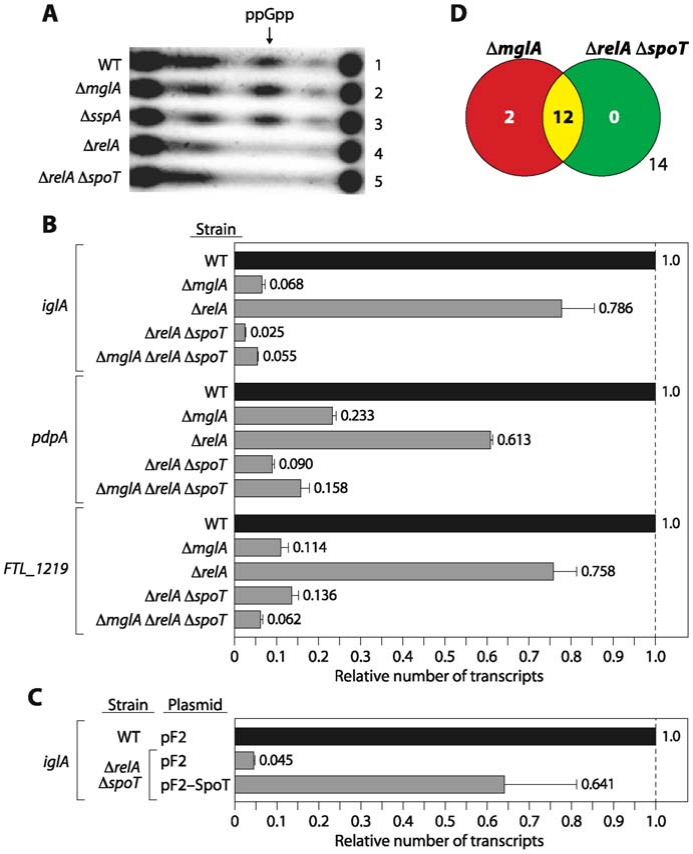
ppGpp controls the expression of MglA/SspA-regulated genes in *F. tularensis*. (A) Analysis of ppGpp concentrations in cells of the indicated strains of LVS by thin layer chromatography. For these analyses ppGpp was isolated from cells following a shift to conditions of nutrient limitation (see Materials and Methods). (B) Quantitative RT-PCR analysis of *iglA*, *pdpA*, and *FTL_1219* transcript abundance in wild-type (WT), Δ*mglA*, Δ*relA*, Δ*relA* Δ*spoT*, and Δ*mglA* Δ*relA* Δ*spoT* mutant backgrounds. RNA was isolated from cells grown in MH to mid-log. Transcripts were normalized to those of *tul4*, whose expression is not influenced by MglA or ppGpp. (C) Complementation of the effects of the Δ*relA* Δ*spoT* mutations on *iglA* expression by *spoT* provided in trans. Quantitative RT-PCR analysis of *iglA* transcript abundance in wild-type (WT), and Δ*relA* Δ*spoT* mutant cells harboring the indicated plasmids. Transcripts were normalized to *tul4*. Plasmid pF2-SpoT directs the synthesis of *F. tularensis* SpoT, whereas plasmid pF2 served as an empty vector control. (D) Venn diagram representation of the overlap between genes controlled by MglA and ppGpp. Each circle represents those genes whose expression was decreased by a factor of 2.5 or more (p<0.05) in the indicated mutant background compared to wild-type and whose expression altered by a factor of 2 or more in the other mutant background, as determined by DNA-microarray.

In the course of testing specific models for how the MglA-SspA complex controls virulence gene expression we uncovered a critical role for the alarmone ppGpp. To test whether ppGpp might influence the expression of MglA/SspA-regulated genes we constructed derivatives of the live vaccine strain of *F. tularensis* (LVS) (an attenuated derivative of an *F. tularensis* subspecies *holarctica* strain) carrying in-frame deletions of the *relA* gene (LVS Δ*relA*), the *relA* and *spoT* genes (LVS Δ*relA* Δ*spoT*), and the *mglA*, *relA*, and *spoT* genes (LVS Δ*mglA* Δ*relA* Δ*spoT*); as in other Gram-negative bacteria, deletion of *relA* and *spoT* in LVS resulted in a ppGpp null mutant (ppGpp°) that no longer makes detectable amounts of ppGpp (Figure 1A). To determine whether deletion of *relA*, or deletion of *relA* and *spoT*, had any effect on the expression of MglA/SspA-regulated genes, RNA was isolated from mid-log grown cells of wild-type LVS, LVS Δ*mglA*, LVS Δ*relA*, LVS Δ*relA* Δ*spoT*, and LVS Δ*mglA* Δ*relA* Δ*spoT*, and expression of the MglA/SspA-regulated *iglA*, *pdpA*, and *FTL_1219* genes was measured by quantitative RT-PCR (qRT-PCR).

Deletion of *mglA* or *relA* and *spoT* caused a similar drastic reduction in the amounts of the *iglA*, *pdpA*, and *FTL_1219* transcripts when compared to LVS wild-type cells (Figure 1B). Furthermore, similar amounts of the *iglA*, *pdpA*, and *FTL_1219* transcripts were seen in cells of the Δ*mglA* mutant, the Δ*relA* Δ*spoT* mutant, and the Δ*mglA* Δ*relA* Δ*spoT* mutant. Only slightly reduced amounts of the *iglA*, *pdpA*, and *FTL_1219* transcripts were present in LVS Δ*relA* mutant cells relative to wild-type cells. Complementation of LVS Δ*relA* Δ*spoT* mutant cells with a plasmid expressing *spoT* restored the amounts of the *iglA* transcript close to wild-type or Δ*relA* mutant levels (Figure 1C). Because the LVS Δ*relA* Δ*spoT* mutant can no longer make ppGpp, we infer from our results that ppGpp positively regulates the expression of MglA/SspA-regulated genes located both within (*iglA* and *pdpA*), and outside of (*FTL_1219*), the FPI. Moreover, MglA and ppGpp appear to exert similar effects on target gene expression. In the case of two of these target genes, deletion of *mglA* in a Δ*relA* Δ*spoT* mutant background did not result in a further decrease in expression suggesting that the effects of MglA and ppGpp are dependent on one another.

To determine whether ppGpp and the MglA-SspA complex regulate expression of the same set of genes, we performed DNA microarray analyses to compare the global gene expression profiles of Δ*mglA* mutant cells, Δ*relA* Δ*spoT* mutant cells, and LVS wild-type cells. The results of these analyses reveal that in LVS, ppGpp and MglA regulate expression of essentially the same set of genes (Figure 1D; Table S1).

### A Δ*relA* Δ*spoT* mutant is defective for intramacrophage growth and for virulence in mice

**Figure 2:**
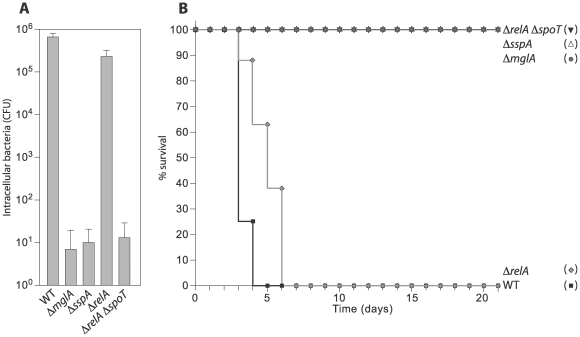
Cells of a Δ*relA* Δ*spoT* mutant are defective for intramacrophage growth and for virulence in mice. (A) Survival of wild-type (WT) and the indicated mutant derivates of *F. tularensis* strain LVS within J774 cells. J774 murine macrophages were infected with cells of the indicated bacterial strains at a multiplicity of infection of ∼15. Cells were lysed and bacteria (colony forming units [CFU]) were plated for enumeration 24 hours post-infection. (B) Survival of BALB/cByJ mice following intradermal delivery of ∼10^7^ cells of each of the indicated strains of LVS. Eight mice were inoculated for each strain tested. Experiments were performed at least twice.

MglA is thought to be essential for intramacrophage growth at least in part because it positively regulates the expression of genes present on the FPI that are in turn required for intramacrophage growth or survival [4], [18]–[20]. If MglA, SspA, and ppGpp function together to positively regulate the same set of genes, including many required for virulence, then we would predict that like MglA [4],[18],[19],[47], SspA and ppGpp should be required for intramacrophage growth and for virulence in mice. To test this prediction, we compared the abilities of LVS Δ*mglA*, LVS Δ*sspA*, LVS Δ*relA*, LVS Δ*relA* Δ*spoT* mutant cells, and LVS wild-type cells to replicate within J774 murine macrophages and to cause lethality in BALB/cByJ mice. Approximately 10^5^-fold fewer cells of the LVS Δ*mglA* mutant, the LVS Δ*sspA* mutant, and the LVS Δ*relA* Δ*spoT* mutant were recovered from J774 cells 24 hours post-infection compared to wild-type cells (Figure 2A), and cells of the LVS Δ*mglA*, LVS Δ*sspA*, and LVS Δ*relA* Δ*spoT* mutants were essentially avirulent in BALB/cByJ mice (Figure 2B). Furthermore, in accordance with our finding that *relA* has only a modest effect on the expression of MglA/SspA-regulated genes in broth-grown cells, Δ*relA* mutant cells were only marginally defective for intramacrophage growth and were highly virulent in our mouse model (Figure 2). These findings suggest that, like MglA and SspA, ppGpp is essential for intramacrophage growth and for virulence in mice and are consistent with the idea that the MglA-SspA complex and ppGpp play important roles in positively regulating the expression of virulence genes in *F. tularensis*.

### ppGpp has no effect on the abundance of MglA or SspA, and does not influence the association of the MglA-SspA complex with RNAP

**Figure 3:**
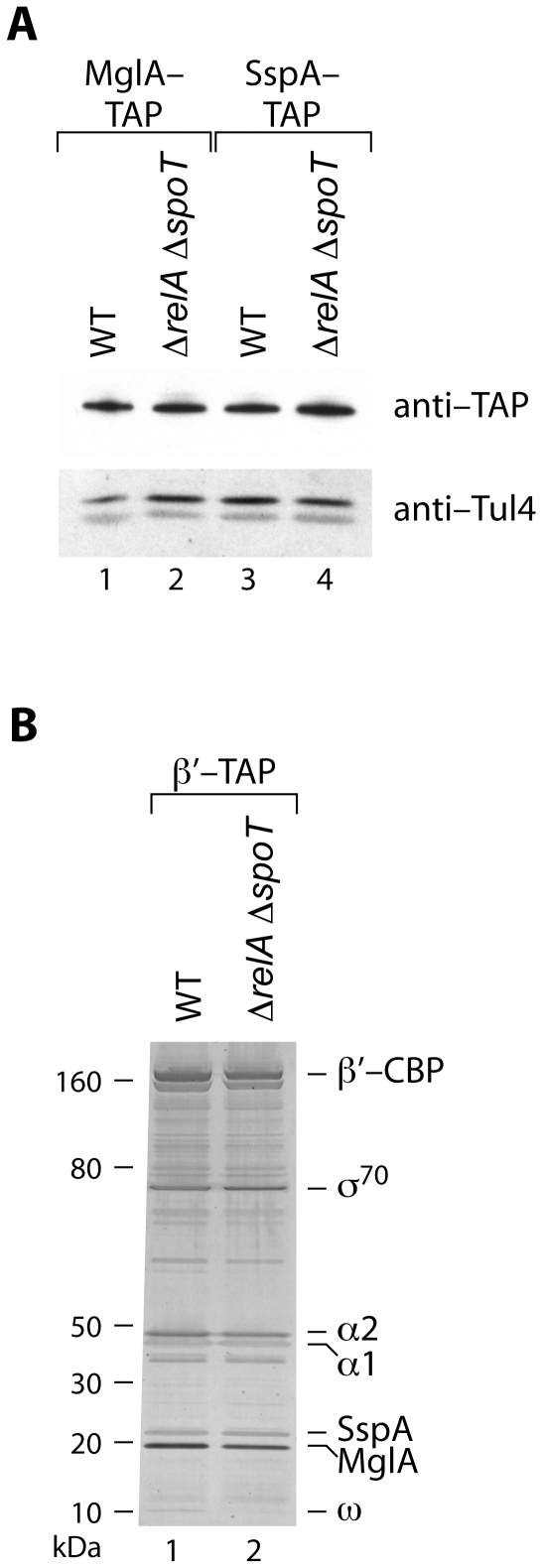
ppGpp does not influence the abundance of MglA or SspA, and does not influence the association between RNAP and the MglA-SspA complex. (A) Western blot analysis of the effect of ppGpp on the abundance of MglA-TAP and SspA-TAP. Proteins from equivalent numbers of LVS MglA-TAP cells (lane 1), LVS Δ*relA* Δ*spoT* MglA-TAP cells (lane 2), LVS SspA-TAP cells (lane 3), and LVS Δ*relA* Δ*spoT* SspA-TAP cells (lane 4) were electrophoresed on a 4–12% Bis-Tris NuPAGE gel and analysed by Western blotting. Upper panel, immunoblot probed with anti-TAP. Lower panel, immunoblot probed with antibody against Tul4 serves as a control for sample loading. (B) SDS-PAGE analysis of proteins that co-purify with β′-TAP in a wild-type (WT, lane 1) or in a Δ*relA* Δ*spoT* mutant background (lane 2). Protein complexes were tandem affinity purified, electrophoresed on a 4–12% Bis-Tris NuPAGE gel, and stained with silver. Lane 1, proteins purified from strain LVS β′-TAP. Lane 2, proteins purified from strain LVS Δ*relA* Δ*spoT* β′-TAP. Molecular weights are indicated on the left. The identities of certain proteins are indicated on the right.

In *E. coli*, *relA* is required for the synthesis of SspA in stationary phase cells, presumably because ppGpp is required for expression of the *sspA* gene [23]. This raises the possibility that in *F. tularensis* ppGpp might influence the expression of MglA/SspA-regulated genes through an effect on the expression of *mglA* or *sspA*. However, microarray and qRT-PCR analyses revealed that there was a less than two-fold difference in the abundance of the *mglA* and *sspA* transcripts in cells of the wild-type strain relative to those of the Δ*relA* Δ*spoT* mutant, suggesting that ppGpp does not significantly influence expression of the *sspA* or *mglA* genes (data not shown). To determine whether ppGpp influences the abundance of the MglA or SspA proteins we used derivatives of wild-type LVS and LVS Δ*relA* Δ*spoT* in which the native chromosomal copies of *mglA* and *sspA* have been altered such that they specify forms of MglA or SspA with C-terminal tandem affinity purification (TAP) tags (LVS MglA-TAP, LVS SspA-TAP, LVS Δ*relA* Δ*spoT* MglA-TAP, and LVS Δ*relA* Δ*spoT* SspA-TAP) [20]. Comparison of the amounts of MglA-TAP and SspA-TAP in wild-type and Δ*relA* Δ*spoT* mutant cells, as revealed by Western blotting, suggests that ppGpp does not have any positive effect on the abundance of MglA-TAP or SspA-TAP (Figure 3A).

Although ppGpp does not appear to influence the amount of MglA or SspA in the cell, ppGpp could control expression of MglA/SspA-regulated genes through an effect on the association of the MglA-SspA complex with RNAP. To test this possibility we used derivatives of wild-type LVS and LVS Δ*relA* Δ*spoT* that contain a TAP-tagged form of the β′ subunit of RNAP (LVS β′-TAP and LVS Δ*relA* Δ*spoT* β′-TAP). Following purification of RNAP and associated proteins from these strains by TAP, proteins were separated by SDS-PAGE and stained with silver. These analyses revealed that ppGpp does not appear to influence the ability of the MglA-SspA complex to associate with RNAP, i.e. the MglA-SspA complex is associated with RNAP regardless of the presence or absence of ppGpp (Figure 3B). Because MglA does not associate with RNAP in the absence of SspA [20], these findings suggest furthermore that ppGpp does not prevent MglA from interacting with SspA. Although it was formally possible that MglA and SspA could be required for the synthesis of ppGpp, thin layer chromatography of ^32^P-labelled nucleotides isolated from LVS wild-type, Δ*mglA* mutant, and Δ*sspA* mutant cells suggests that this is not the case (Figure 1A).

### Genetic screen identifies CaiC, TrmE, CphA, and FTL_0449 (PigR) as positive regulators of MglA/SspA-regulated genes in *F. tularensis*

**Figure 4:**
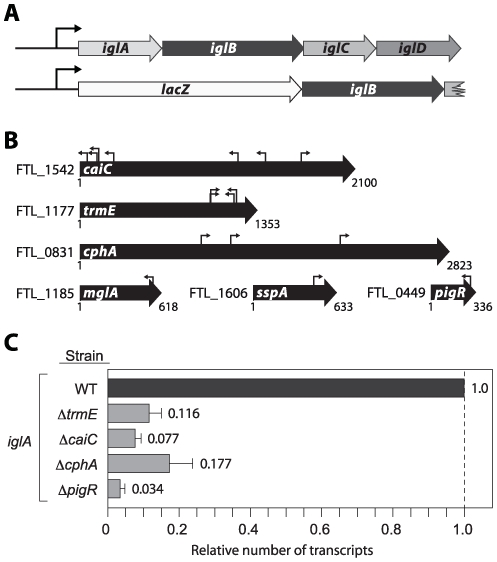
Genetic screen for positive regulators of *iglA* expression. (A) Schematic of *iglA* reporter in strain LVS *iglA::lacZ* in which one of the copies of the *iglA* gene has been replaced with *lacZ*. (B) Location of transposon insertions (indicated by small arrows) in genes identified in the screen. Numbers below each gene refer to base pairs. (C) Quantitative RT-PCR analysis of *iglA* transcript abundance in wild-type (WT), Δ*trmE*, Δ*caiC*, Δ*cphA*, and Δ*pigR* mutant backgrounds. Transcripts were normalized to those of *tul4*.

Given that ppGpp does not influence the abundance of MglA or SspA, and does not prevent association of the MglA-SspA complex with RNAP, we postulated that ppGpp might exert its effects through another regulator, distinct from MglA and SspA, that in turn is required for expression of the MglA/SspA regulon. In an attempt to identify such a regulator we took an unbiased genetic approach. We constructed a reporter strain in which the *iglA* gene on one of the copies of the FPI was replaced with *lacZ* (LVS Δ*iglA::lacZ*) (Figure 4A); expression of *iglA* is strongly dependent on MglA and SspA [20]. We then mutagenized the LVS Δ*iglA::lacZ* reporter strain with a mariner transposon and screened for mutants that displayed decreased levels of *lacZ* expression on CHA agar plates containing X-Gal. Among the transposon mutants with decreased β-galactosidase activity relative to the non-mutagenized reporter strain were isolates containing transposons in *mglA*, *sspA*, *caiC* (encoding a putative acyl-CoA synthetase), *cphA* (encoding a putative cyanophycin synthetase), *trmE* (encoding a putative GTPase with methyltransferase activity), and *FTL_0449* (encoding a putative DNA-binding protein with similarity to members of the MerR family of transcription regulators) (Figure 4B). Because we originally identified FTL_0449 as a positive regulator of a gene present on the FPI, we have named FTL_0449 PigR, for pathogenicity island gene regulator.

To confirm that inactivation of each of the genes identified in the screen was responsible for the effect of the original transposon insertions on *iglA* expression, we made mutants of LVS carrying in-frame deletions of the *caiC*, *trmE*, *cphA*, and *pigR* (*FTL_0449*) genes. We then quantified *iglA* transcript levels in cells of each of these mutant strains, and in LVS wild-type cells by qRT-PCR. Deletion of *caiC*, *trmE*, *cphA*, or *pigR* resulted in large decreases in the amount of the *iglA* transcripts (Figure 4C). Complementation of cells of the LVS Δ*caiC*, LVS Δ*trmE*, LVS Δ*cphA*, and LVS Δ*pigR* mutant strains with plasmids expressing either *caiC*, *trmE*, *cphA*, or *pigR*, respectively, restored the amounts of *iglA* transcripts close to wild-type levels (Figure S1). Thus, CaiC, TrmE, CphA, and PigR positively regulate expression of the FPI-encoded *iglA* gene. In addition, DNA microarray analyses revealed that CaiC, TrmE, CphA, and PigR control the expression of the entire MglA/SspA regulon (Table S1). Taken together, our findings suggest that a common set of target genes are positively regulated by CaiC, TrmE, CphA, PigR, ppGpp and MglA/SspA.

### PigR and TrmE are required for intramacrophage growth and for virulence in mice

**Figure 5:**
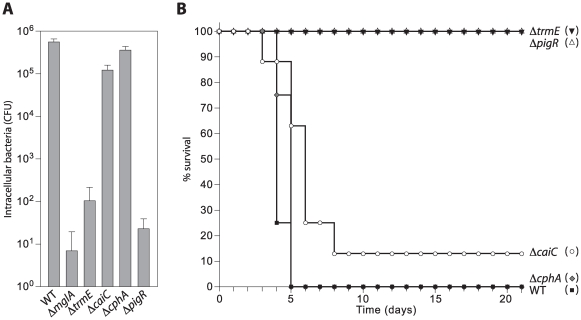
PigR and TrmE are required for intramacrophage growth and for virulence in mice. (A) Survival of wild-type (WT) cells, and cells of the indicated mutant derivates of *F. tularensis* strain LVS within J774 cells. J774 murine macrophages were infected with cells of the indicated bacterial strains at a multiplicity of infection of ∼15. Cells were lysed and bacteria (colony forming units [CFU]) were plated for enumeration 24 hours post-infection. (B) Survival of BALB/cByJ mice following intradermal delivery of ∼10^7^ cells of each of the indicated strains of LVS. Eight mice were inoculated for each strain tested. Experiments were performed at least twice.

We next compared the abilities of LVS Δ*caiC* mutant cells, LVS Δ*trmE* mutant cells, LVS Δ*cphA* mutant cells, LVS Δ*pigR* mutant cells, and LVS wild-type cells to replicate within macrophages and to cause virulence in mice. We infer from the results depicted in Figure 5A that in LVS, only TrmE and PigR are required for intramacrophage growth or survival. Furthermore, consistent with these findings, only the Δ*trmE* mutant cells, and the Δ*pigR* mutant cells are avirulent in mice (Figure 5B). These findings suggest that both TrmE and PigR are critical regulators of virulence gene expression in vivo, and that although CaiC and CphA play a significant role in controlling the expression of MglA/SspA regulated genes in broth-grown cells, they may not significantly influence the expression of these genes in vivo, at least not in the infection models used here.

### ppGpp positively regulates *pigR* expression and may function upstream of PigR and MglA/SspA

**Figure 6:**
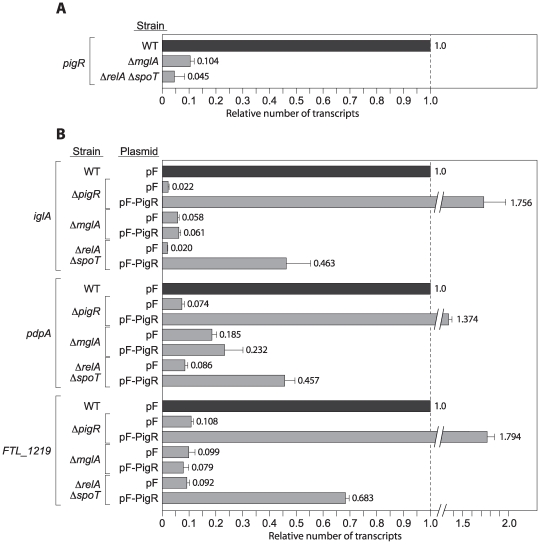
Ectopic expression of *pigR* partially complements a Δ*relA* Δ*spoT* mutant but fails to complement a Δ*mglA* mutant. (A) Expression of *pigR* is positively regulated by MglA and by ppGpp. Quantitative RT-PCR analysis of *iglA* transcript abundance in wild-type (WT), Δ*mglA*, and Δ*relA* Δ*spoT* mutant backgrounds. Transcripts were normalized to those of *tul4*. (B) Quantitative RT-PCR analysis of *iglA*, *pdpA*, and *FTL_1219* transcript abundance in wild-type (WT), Δ*pigR*, Δ*mglA*, and Δ*relA* Δ*spoT* mutant cells containing the indicated plasmids. Plasmid pF-PigR directs the synthesis of PigR and plasmid pF serves as an empty vector control. Transcripts were normalized to those of *tul4*.

Amongst the regulators identified in our screen, only PigR appeared to be subject to control by ppGpp; *pigR* is amongst the genes that we identified as being under the control of ppGpp in our DNA microarray analyses (Table S1), a finding we confirmed by using qRT-PCR to quantify the abundance of the *pigR* transcript in wild-type and Δ*relA* Δ*spoT* mutant cells (Figure 6A). However, *pigR* (*FTL_0449*) is also amongst the genes that we identified previously as being under the control of MglA and SspA in LVS [20] (and confirmed using qRT-PCR in Figure 6A). These observations raise the possibility that the MglA-SspA complex and ppGpp might serve simply to control *pigR* expression, and that PigR itself might be directly responsible for regulating the expression of target genes. This model, in which MglA/SspA and ppGpp function upstream of PigR, predicts that ectopic expression of *pigR* should complement the phenotypes of a Δ*mglA* mutant, and a Δ*relA* Δ*spoT* mutant.

To determine where PigR operates in the regulatory hierarchy, we introduced a vector expressing *pigR* (pF-PigR) and an empty control vector (pF), into LVS Δ*mglA*, LVS Δ*relA* Δ*spoT*, and LVS Δ*pigR* mutant cells. (Expression of *pigR* is under the control of the heterologous *groEL* promoter on pF-PigR.) As a control, we also introduced the empty vector into wild-type LVS. Cells containing these plasmids were grown to mid-log, RNA was isolated, and the expression of the *iglA*, *pdpA*, and *FTL_1219* transcripts was quantified by qRT-PCR.

Ectopic expression of *pigR* (i) restored expression of the MglA/SspA-regulated genes to above wild-type levels in cells of the Δ*pigR* mutant strain, (ii) failed to restore expression of the MglA/SspA-regulated genes in Δ*mglA* mutant cells, and (iii) only partially restored expression of the MglA/SspA-regulated genes in cells of the Δ*relA* Δ*spoT* mutant strain (Figure 6B). These findings suggest that PigR functions together with the MglA-SspA complex downstream of ppGpp.

### PigR autoactivates

Expression of the *pigR* gene is positively controlled by the MglA-SspA complex. If, as is suggested by our findings, PigR functions together with the MglA-SspA complex then we would predict that the expression of the *pigR* gene is positively regulated by PigR. ppGpp also regulates expression of *pigR*. Conceivably, by regulating expression of *pigR* (independently of PigR and the MglA-SspA complex), ppGpp might drive expression of sufficient amounts of *pigR* to promote autoactivation. This led us to test the prediction that PigR positively regulates the expression of its own gene (in concert with MglA and SspA), and to test the idea that ppGpp serves simply to positively regulate the expression of the *pigR* gene.

**Figure 7:**
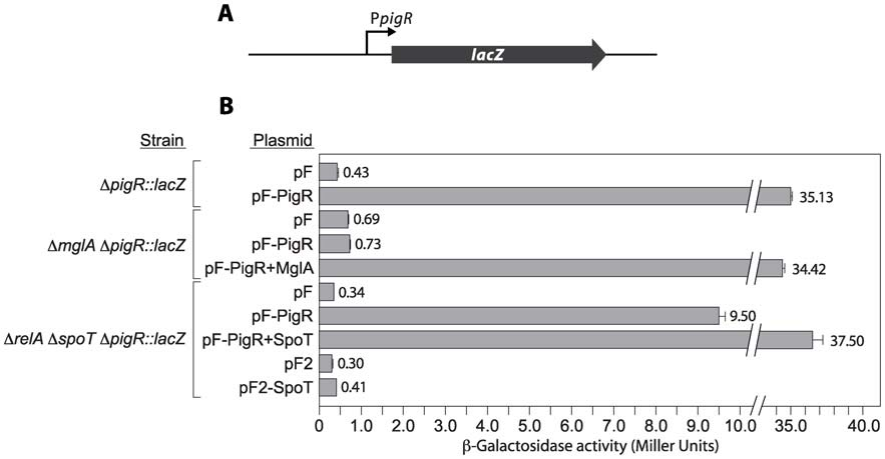
The *pigR* gene is positively autoregulated. (A) Schematic of *lacZ* reporter in strain LVS *pigR::lacZ*. In this strain the native *pigR* gene was replaced with *lacZ*, thus placing *lacZ* under the control of the *pigR* promoter (P*pigR*) on the LVS chromosome. (B) Quantification of *lacZ* expression in strains LVS *pigR::lacZ*, LVS Δ*mglA pigR::lacZ*, and LVS Δ*relA* Δ*spoT pigR::lacZ* containing the indicated plasmids. Plasmids pF-PigR, pF-PigR+MglA, pF-PigR+SpoT, and pF2-SpoT direct the synthesis of PigR, PigR and MglA, PigR and SpoT, and SpoT, respectively. Plasmid pF served as the empty control vector for plasmids pF-PigR, pF-PigR+MglA, and pF-PigR+SpoT, whereas plasmid pF2 served as the empty control vector for plasmid pF2-SpoT.

To determine whether PigR positively regulates the expression of its own gene we constructed a strain of LVS that contains *lacZ* in place of *pigR* (LVS *pigR::lacZ*) (Figure 7A). Ectopic expression of *pigR* (from plasmid pF-PigR that has *pigR* situated downstream of the heterologous *groEL* promoter) increased β-galactosidase activity in LVS *pigR*::*lacZ* ∼100-fold, indicating that PigR autoactivates (Figure 7B). This finding is consistent with the idea that PigR functions coordinately with the MglA-SspA complex to control the expression of all target genes.

Using a Δ*relA* Δ*spoT* mutant derivative of the Δ*pigR::lacZ* reporter strain (LVS Δ*relA* Δ*spoT* Δ*pigR::lacZ*) we found that ppGpp did not detectably influence the basal activity of the *pigR* promoter in the absence of PigR (Figure 7B), suggesting that the effect of ppGpp is dependent upon the presence of PigR. Furthermore, we found that although ectopic expression of *pigR* resulted in an ∼30-fold increase in expression of the *pigR* reporter in the Δ*relA* Δ*spoT* mutant strain, it resulted in an ∼110-fold increase in expression of the reporter in the same Δ*relA* Δ*spoT* mutant strain when *spoT* was also provided ectopically (Figure 7B), indicating that ectopic expression of *pigR* from a heterologous promoter can only partially alleviate the defects of a Δ*relA* Δ*spoT* mutant. Taken together, our findings suggest that expression of the *pigR* gene is subject to autoactivation, that PigR and the MglA-SspA complex function coordinately to regulate the expression of all target genes (including *pigR*), and either that ppGpp influences the activity of PigR, or that ppGpp and PigR act by independent mechanisms but are both needed for *pigR* expression.

### PigR interacts with the MglA-SspA complex

Most classical transcription activators function by binding the DNA and contacting a particular subunit of RNAP [48]. Because PigR works in concert with the MglA-SspA complex, and because the MglA-SspA complex associates with RNAP [20], we hypothesized that PigR may activate transcription in *F. tularensis* through a direct contact with the RNAP-associated MglA-SspA complex.

In order to test whether PigR can interact directly with the MglA-SspA complex we used a bacterial two-hybrid assay. This two-hybrid assay is based on the finding that any sufficiently strong interaction between two proteins can activate transcription in *E. coli* provided one of the interacting proteins is tethered to the DNA by a DNA-binding protein and the other is tethered to a subunit of *E. coli* RNAP [49],[50]. In the version of the assay used here, contact between a protein (or protein domain) fused to the ω subunit of *E. coli* RNAP and another protein fused to a zinc-finger DNA-binding protein (referred to as Zif) activates transcription of a *lacZ* reporter gene situated downstream of an appropriate test promoter containing a Zif binding site [51].

**Figure 8:**
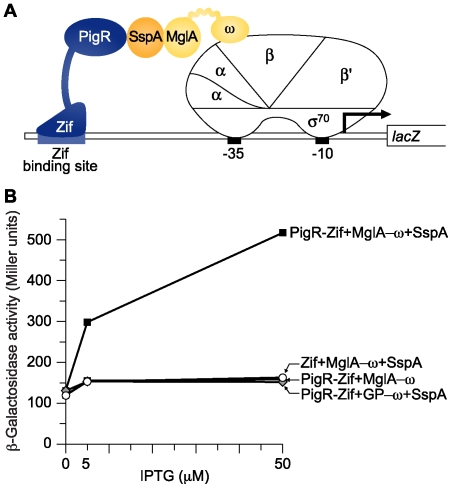
PigR interacts with the MglA-SspA complex. (A) Schematic representation of the bacterial bridge-hybrid system used to detect an interaction between PigR and the MglA-SspA complex. In this system *F. tularensis* SspA interacts with the MglA-ω fusion protein to form a heteromeric complex that associates with *E. coli* RNAP. Contact between the heteromeric MglA-SspA complex displayed on RNAP and the DNA-bound PigR-Zif fusion activates transcription from the test promoter driving expression of *lacZ*. The test promoter p*lac*Zif1-61 is present on an F′ episome in *E. coli* strain KDZif1ΔZ and bears a Zif binding site centered 61 bp upstream of the transcription start site of the *lac* core promoter (whose −10 and −35 elements are indicated). (B) Transcription activation by PigR-Zif in the presence of *F. tularensis* SspA and the MglA-ω fusion protein. Assays were performed with cells of the *E. coli* reporter strain KDZif1ΔZ containing compatible plasmids that directed the IPTG-controlled synthesis of the indicated proteins. Cells were grown in the presence of different concentrations of IPTG and assayed for β-galactosidase activity.

Our strategy for detecting a putative interaction between PigR and the MglA-SspA complex required that we modify the two-hybrid assay to permit detection of an interaction between PigR and a heteromeric complex. We reasoned that if we made *F. tularensis* SspA in our *E. coli* reporter strain, together with a fusion protein in which PigR was fused to Zif (PigR-Zif), and another in which MglA was fused to ω (MglA-ω), SspA would interact with the MglA-ω fusion protein, presenting a heteromeric target on RNAP that could be bound by the PigR moiety of the PigR-Zif fusion protein (Figure 8A). Therefore, for these “bridge-hybrid” experiments, plasmids directing the synthesis of a PigR-Zif fusion protein, an MglA-ω fusion protein [20], and *F. tularensis* SspA were introduced into the *E. coli* reporter strain KDZif1ΔZ, which harbors the test promoter depicted in Figure 8A linked to *lacZ* on an F′ episome [20],[51]. In support of the idea that PigR interacts with the MglA-SspA complex directly, the PigR-Zif fusion protein activated transcription from the test promoter only in the presence of both SspA and the MglA-ω fusion (Figure 8B).

### Evidence that ppGpp promotes the interaction between PigR and the MglA-SspA complex

Our findings suggest that PigR and the MglA-SspA complex function together to regulate the expression of target genes through a direct contact and that ppGpp might be required for the optimal activity of PigR. We therefore wondered whether ppGpp might exert its effects by modulating the interaction between PigR and the MglA-SspA complex.

**Figure 9:**
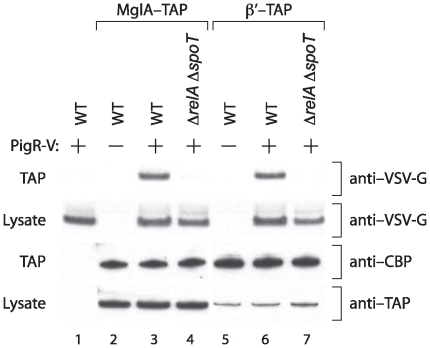
ppGpp promotes the interaction between PigR and the MglA-SspA complex. PigR-V was ectopically expressed in wild-type (WT) or in Δ*relA* Δ*spoT* mutant cells producing MglA-TAP, or β′-TAP. Cells were grown to mid-log and formaldehyde was added to crosslink PigR-V to associated proteins. Following TAP and reversal of the crosslinks, proteins that co-purified with MglA-TAP, or β′-TAP were separated on a 4–12% Bis-Tris NuPAGE gel and the presence of PigR-V was analyzed by Western blotting. Proteins present in cell lysates prior to TAP were separated and analyzed in the same manner. Upper panels: PigR-V present following TAP (top), and PigR-V present in the cell lysate prior to TAP (bottom). Lower panels: Calmodulin binding peptide (CBP)-tagged proteins present following TAP (top), and TAP-tagged proteins present in the cell lysate prior to TAP (bottom). Note that because the protein A moieties of the TAP-tag are removed during the tandem affinity purification procedure, the CBP portion of the TAP-tag is all that remains following TAP. The anti-CBP antibody is therefore used here to detect proteins that initially contained the TAP-tag. Note also that the MglA and β′ species are different sizes and so different portions of the corresponding Western blot are shown. Proteins were from the following cells: Lane 1, LVS synthesizing PigR-V. Lane 2, LVS MglA-TAP. Lane 3, LVS MglA-TAP synthesizing PigR-V. Lane 4, LVS MglA-TAP Δ*relA* Δ*spoT* synthesizing PigR-V. Lane 5, LVS β′-TAP. Lane 6, LVS β′-TAP synthesizing PigR-V. Lane 7, LVS β′-TAP Δ*relA* Δ*spoT* synthesizing PigR-V.

To determine whether ppGpp might influence the ability of PigR to interact with the MglA-SspA complex we took an in vivo crosslinking approach. First we constructed an epitope-tagged version of PigR that contained a vesicular stomatitis virus glycoprotein (VSV-G) epitope-tag fused to its C-terminus. The resulting fusion protein (PigR-V) was functional as it could complement a Δ*pigR* mutant strain (see Figure S2). We then ectopically expressed *pigR-V* from a heterologous promoter in cells of LVS, LVS MglA-TAP, and LVS Δ*relA* Δ*spoT* MglA-TAP. Cells of the LVS MglA-TAP strain that did not express *pigR-V* served as an additional control. Formaldehyde was added to cells in order to crosslink PigR-V to the MglA-SspA complex. Following TAP, reversal of the crosslinks, and separation of proteins by SDS-PAGE, the amount of PigR-V associated with MglA was assessed by Western blotting. The results depicted in Figure 9 show that considerably less PigR-V was found associated with MglA-TAP in cells of the Δ*relA* Δ*spoT* mutant, than in cells of the corresponding wild-type strain, despite there being similar amounts of PigR-V or MglA-TAP present in each cell type. Furthermore, the observed association between PigR and MglA was resistant to DNase treatment (data not shown). These findings suggest that ppGpp stimulates the protein-protein interaction between PigR and the MglA-SspA complex. To address whether ppGpp influences the interaction between PigR and MglA-SspA complexes that are associated with RNAP we performed a similar set of in vivo crosslinking experiments with cells of our LVS β′-TAP and LVS Δ*relA* Δ*spoT* β′-TAP strains. The results depicted in Figure 9 suggest that PigR associates with RNAP and that ppGpp influences this association. Thus, in *Francisella* ppGpp appears to exert its effects on gene expression by promoting the interaction between PigR and the RNAP-associated MglA-SspA complex.

## Discussion

The SspA protein family members MglA and SspA are key regulators of virulence gene expression in *F. tularensis*. We have found that the alarmone ppGpp and the putative DNA-binding protein, PigR, function in concert with MglA and SspA to regulate a common set of genes. Consistent with this idea, we have shown that cells of both a Δ*pigR* mutant strain and a Δ*relA* Δ*spoT* mutant strain fail to replicate inside macrophages and are severely attenuated in a mouse model of infection, just like cells of a Δ*mglA* or a Δ*sspA* mutant. Furthermore, we have uncovered the molecular basis for the coordinate activities of PigR, ppGpp, and the MglA-SspA complex; we have shown that PigR interacts directly with the MglA-SspA complex, and presented evidence that ppGpp modulates this interaction.

### PigR functions coordinately with the MglA-SspA complex

We identified PigR through a genetic screen for positive regulators of MglA/SspA-controlled genes in LVS. PigR resembles a classical transcription regulator as it may contain a helix-turn-helix (HTH) motif (residues 33–54), and shares a limited degree of homology with members of the MerR family of transcription regulators. Note that the ligand-binding or co-activator-binding C-terminal domain, which is often found in MerR family members [52], does not appear to be present in PigR. PigR was also recently identified in a screen for regulators of MglA/SspA-regulated genes in *F. novicida*, where it is called FevR [15]. As is the case for PigR in LVS, FevR in *F. novicida* is essential for replication in macrophages and for virulence in mice [15]. Our demonstration that PigR interacts with the MglA-SspA complex provides a molecular explanation for the findings that PigR in LVS, and FevR in *F. novicida* [15], control the expression of all MglA/SspA-regulated genes.

**Figure 10:**
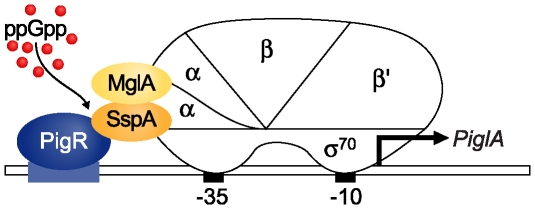
Model for how the MglA-SspA complex, ppGpp, and PigR, positively control the expression of target genes. According to our model PigR interacts directly with the RNAP-associated MglA-SspA complex and ppGpp promotes this interaction (either by interacting directly with PigR and/or the MglA-SspA complex, or by interacting directly with RNAP). Although the MglA-SspA complex is depicted as interacting with the α subunit of RNAP (for convenience), it is not known which RNAP subunit(s) serve as a contact site for the MglA-SspA complex.

Our model for how ppGpp, the MglA-SspA complex, and PigR collaborate to control the expression of target genes is depicted schematically in Figure 10. The model specifies that PigR is a transcription activator whose activation target on RNAP is the MglA-SspA complex. Target promoters would contain specific binding sites for PigR, and contact between PigR and the RNAP-associated MglA-SspA complex would stabilize the binding of RNAP to the promoter, thereby activating transcription. Thus, in this model, promoter specificity is determined by the DNA sequence-specific binding of PigR, and the MglA-SspA complex effectively becomes a subunit of RNAP that serves as a contact site for DNA-bound PigR (Figure 10). The model further specifies that ppGpp exerts its effects by positively influencing the interaction between PigR and the MglA-SspA complex (Figure 10), through a mechanism that has yet to be elucidated. Note that in this model, PigR and MglA/SspA-regulated promoters are depicted as being recognized by RNAP holoenzyme containing σ^70^. Consistent with the idea that these promoters are σ^70^-dependent, the RNAP that copurifies with MglA appears to contain σ^70^ in stoichiometric amounts [20]. Variations on this model are also consistent with our findings and it remains to be determined whether PigR is a DNA-binding protein that recognizes specific sites at target promoters.

Our studies of PigR are the first to demonstrate a direct interaction between a transcription activator and members of the SspA protein family. However, the sequence-specific DNA-binding protein Lpa from bacteriophage P1 may function analogously to PigR and activate transcription from target promoters by making simultaneous contact with the DNA and with the *E. coli* RNAP-associated SspA protein. Indeed, *E. coli* SspA can function as a co-activator of bacteriophage P1 late gene expression [53].

### ppGpp promotes the interaction between PigR and the MglA-SspA complex

Our findings suggest that in *F. tularensis*, ppGpp exerts its effects on transcription at multiple different promoters by promoting the interaction between PigR and the MglA-SspA complex. In *E. coli*, and a few other Gram-negative bacteria where it has been studied in any detail, ppGpp influences transcription at multiple promoters through a direct interaction with RNAP [28]. In particular, by interacting with RNAP, ppGpp can exert direct inhibitory or stimulatory effects on transcription initiation at many different promoters, typically in combination with the RNAP-associated DksA protein [28],[32]. In these instances the specific kinetic parameters of the target promoters render them responsive to ppGpp [28]. Conceivably, an interaction between ppGpp and *F. tularensis* RNAP may influence the binding of PigR to the RNAP-associated MglA-SspA complex through an allosteric mechanism. Alternatively, ppGpp might mediate its effects by interacting directly with PigR or the MglA-SspA complex, or both. In support of such a notion, several proteins other than RNAP are thought to bind ppGpp directly [30],[54]. It is therefore possible that the basis for the global effects of ppGpp on transcription in *F. tularensis* differs fundamentally from that in *E. coli*.

We do not yet know how ppGpp influences the interaction between PigR and the MglA-SspA complex. However, in considering potential mechanisms for how this might occur it is worth noting that ppGpp need not necessarily be the molecule directly mediating an effect on gene expression. Because GTP is used up in the process of synthesizing ppGpp [29], it is possible that the observed effects of ppGpp in *F. tularensis* are due to a concomitant decrease in the cellular pools of GTP. Indeed, in *Bacillus subtilis*, the inhibitory effects of ppGpp on rRNA transcription are thought to be an indirect effect of ppGpp on the intracellular concentration of GTP [55].

### The *pigR* gene is positively autoregulated

Expression of the *pigR* gene is itself positively regulated by PigR (in addition to MglA/SspA and ppGpp). This positive feedback loop may serve to amplify the effect of ppGpp on virulence gene expression. The interaction between PigR and the MglA-SspA complex therefore appears to provide an important regulatory checkpoint for the control of virulence gene expression in *F. tularensis*. By being responsive to the signaling molecule ppGpp, this checkpoint presumably serves to relay signals of cellular nutrition and stress to the regulatory network governing virulence gene expression.

### TrmE influences the expression of MglA/SspA-regulated genes

Through a genetic screen for potential regulators of MglA/SspA-regulated genes we identified TrmE, CphA, and CaiC in addition to PigR. CaiC was identified recently in a similar genetic screen in LVS as a regulator of MglA/SspA-controlled genes present on the FPI, and has been renamed MigR [17]. We found that although PigR, CaiC (MigR), CphA, and TrmE influence the expression of all MglA/SspA-regulated genes in broth grown cells, only PigR and TrmE were required for growth in macrophages and for virulence in mice, suggesting that only PigR and TrmE control the expression of MglA/SspA-regulated genes in vivo, at least in the particular infection models used here.

We have yet to determine how TrmE exerts its effects on gene expression. TrmE is a putative GTPase that in other organisms is thought to influence translation through its tRNA methyltransferase activity [56],[57]. In *E. coli*, TrmE has been linked to the regulation of gene expression [56], and in *Streptococcus pyogenes* the TrmE homolog has been shown to regulate virulence gene expression through an effect on translation of the transcription activator RopB [58]. Whether TrmE might mediate its effects on gene expression in *F. tularensis* by influencing the translation of PigR, MglA, SspA, or some other positive regulator of transcription, remains to be seen.

## Materials and Methods

### Ethics statement

All animals were handled in accordance with good animal practice and all work involving animals was approved by the Harvard Institutional Animal Care and Use Committee.

### Plasmids, strains, and growth conditions

*F. tularensis* subspecies *holarctica* strain LVS was provided by Karen Elkins (U.S. Food and Drug Administration, Rockville, MD). Strains LVS *ΔmglA*, LVS Δ*sspA*, LVS MglA-TAP, and LVS β′-TAP have been described previously [20]. All mutant strains and wild-type LVS were grown with aeration at 37°C in modified Mueller-Hinton broth (Difco) supplemented with glucose (0.1%), ferric pyrophosphate (0.025%), and Isovitalex (2%) or on cysteine heart agar (Difco) supplemented with 1% hemoglobin solution (VWR) (CHAH). Under these conditions cells of the wild-type LVS strain had a culture doubling time of ∼162 minutes whereas cells of the LVS Δ*relA* Δ*spoT* mutant strain (see below) had a culture doubling time of ∼168 minutes. When appropriate, kanamycin or nourseothricin (Werner BioAgents) were used for selection at 5 µg/ml. *E. coli* strains XL1-blue (Stratagene) and DH5α F′IQ (Invitrogen) were used as recipients for all plasmid constructions. *E. coli* strain KDZif1ΔZ was used as the reporter strain for the bacterial two-hybrid experiments. KDZif1ΔZ harbors an F′ episome containing the *lac* promoter derivative p*lac*Zif1-61 driving expression of a linked *lacZ* reporter gene and has been described previously [51].

#### Deletion constructs and strains

The allelic replacement vectors used to create in-frame deletion constructs for *relA* (pEX2-*relA*), *spoT* (pEX2-*spoT*), *caiC* (pEX2-*caiC*), *trmE* (pEX2-*trmE*), *pigR* (pEX2-pigR), and *cphA* (pEX2-*cphA*) were generated as described [20]. These vectors were used to create strains LVS Δ*relA*, LVS Δ*caiC*, LVS Δ*trmE*, LVS Δ*pigR*, and LVS Δ*cphA*, respectively, by allelic exchange. Strain LVS Δ*relA* Δ*spoT* was created by using pEX2-*spoT* to delete the *spoT* gene from strain LVS Δ*relA*, and strain LVS Δ*mglA* Δ*relA* Δ*spoT* was created by using pEX2-*mglA* [20] to delete the *mglA* gene from strain LVS Δ*relA* Δ*spoT*. Deletions were confirmed by the PCR.

#### Complementation vectors

Plasmid pF-PigR directs the synthesis of PigR under the control of the *groEL* promoter and was constructed by replacing *gfp* in the plasmid pFNLTP6 *gro*-*gfp* with full-length *pigR* [59]; plasmids pF-PigR-V, pF-PigR+SpoT, and pF-PigR+MglA were made in a similar manner, and express the PigR protein with a VSV-G epitope-tag fused to its C-terminus, the PigR protein together with the SpoT protein, and the PigR protein together with the MglA protein, respectively. Plasmid pF, which has been described previously [20], carries the *groEL* promoter and was used as an empty control vector for complementation experiments. Plasmids pF2-SpoT and pF2-TrmE were used for complementation of the LVS Δ*relA* Δ*spoT* and LVS Δ*trmE* mutant strains, respectively, and were constructed by inserting full-length *spoT* and *trmE* in place of *sspA* in the plasmid pF2-SspA [20]. Expression of the *spoT* and *trmE* genes are therefore under the control of a weakened *groEL* promoter lacking its putative UP-element. Plasmid pF2 [20] contains the same weakened *groEL* promoter that is present on plasmids pF2-SpoT and pF2-TrmE and was used as an empty control vector. Plasmids pCaiC and pCphA were used for complementation of the LVS Δ*caiC* and LVS Δ*cphA* mutant strains, respectively, and were constructed by inserting full-length *caiC* and *cphA*, respectively, together with approximately 100 bp of upstream DNA sequence into plasmid pFNLTP6 [59]; plasmid pFNLTP6 was used as an empty control vector.

#### Plasmids and strains for TAP-tag experiments

Plasmid pEX-MglA-TAP was used to create strains that synthesized a TAP-tagged form of MglA (with a C-terminal TAP-tag) from the native *mglA* locus and has been described previously [20]. Plasmid pEX-RpoC-TAP was used to create strains that synthesized a TAP-tagged form of the β′ subunit of RNAP from the native *rpoC* locus and has been described previously [20]. Plasmid pEX-SspA-TAP was used to create strains that synthesized a TAP-tagged form of SspA from the native *sspA* locus and was made by cloning an ∼400-bp fragment of DNA corresponding to the 3′ portion of *sspA* into the vector pEXTAP [20]; additionally, a weakened *groEL* promoter was inserted in pEX-SspA-TAP upstream of the *sspA* fragment to direct expression of genes downstream of *sspA*-TAP after integration into the *F. tularensis* chromosome. Strain LVS MglA-TAP synthesizes, at native levels, MglA with a TAP-tag fused to its C-terminus and has been described previously [20]. Strain LVS β′-TAP synthesizes, at native levels, the β′-subunit of RNAP with a TAP-tag fused to its C-terminus and has been described previously [20]. Strain LVS SspA-TAP was constructed by electroporating pEX-SspA-TAP into LVS (essentially as described in [20]) and selected on CHAH containing kanamycin. Because pEX-SspA-TAP cannot replicate in *F. tularensis*, only those cells in which the plasmid has integrated into the chromosome can grow on media containing kanamycin. Strains LVS Δ*relA* Δ*spoT* MglA-TAP, LVS Δ*relA* Δ*spoT* SspA-TAP, and LVS Δ*relA* Δ*spoT* β′-TAP were made using plasmids pEX-MglA-TAP, pEX-SspA-TAP, and pEX-RpoC-TAP, respectively, together with strain LVS Δ*relA* Δ*spoT*. For all strains, insertion of the TAP integration vector at the correct chromosomal location was confirmed by the PCR, and production of the corresponding TAP-tagged protein was confirmed by Western blotting with PAP [60], which binds the ProtA moieties of the TAP-tag.

#### Plasmids for bacterial bridge two-hybrid assays

Plasmid pBRGPω directs the synthesis of the Gal11P-ω fusion protein and has been described before [51]. Plasmid pBRGPω confers resistance to carbenicillin, harbors a ColE1 origin of replication, and carries an IPTG-inducible *lac*UV5 promoter that drives expression of the ω fusion protein [51]. Plasmid pBRMglA-ω directs the synthesis of full-length MglA fused to residues 2–90 of *E. coli* ω, is a derivative of plasmid pBRGPω, and has been described previously [20]. The MglA-ω fusion protein is under the control of an IPTG-inducible *lac*UV5 promoter.

Plasmid pACTR-AP-Zif directs the synthesis of Zif, the zinc finger DNA-binding domain of the murine Zif268 protein, and has been described previously [51]. Plasmid pACTR-AP-Zif confers resistance to tetracycline, harbors a p15A origin of replication, and carries an IPTG-inducible *lac*UV5 promoter that drives expression of Zif. Plasmid pACTR-PigR-Zif directs the synthesis of full-length PigR fused to Zif. The pACTR-PigR-Zif fusion plasmid was made by cloning the appropriate NdeI-NotI–digested PCR product into NdeI-NotI–digested pACTR-AP-Zif. The PigR-Zif fusion protein is therefore under the control of the IPTG-inducible *lac*UV5 promoter.

Plasmid pCL1920 [61] directs the synthesis of the LacZα fragment under the control of the *lac* promoter, confers resistance to spectinomycin, and harbors the pSC101 origin of replication. Plasmid pCL-SspA directs the synthesis of full-length, unmodified *F. tularensis* SspA under the control of the IPTG-inducible *lac*UV5 promoter, and was constructed by inserting full-length *sspA* together with the *lac*UV5 promoter into pCL1920.

#### *Francisella* reporter strains

Reporter strains LVS *iglA*::*lacZ* and LVS *pigR*::*lacZ* contain the *E. coli lacZ* gene in place of the LVS *iglA* and *pigR* genes, respectively. The allelic replacement vectors used to create these strains were generated by first amplifying regions flanking the *iglA* and *pigR* genes by the PCR and then splicing the flanking regions to the *E. coli lacZ* gene by overlap-extension PCR. The resulting PCR products were cloned into the suicide plasmid pEX18km [20], yielding plasmids pEX-*iglA*::*lacZ* and pEX-*pigR*::*lacZ*; the constructs were designed so that the *iglA* and *pigR* ORFs were replaced exactly with the *lacZ* ORF. Because the *sacB* gene on pEX18km is insufficient to mediate efficient sucrose counterselection in LVS, a second copy of *sacB* was subcloned from plasmid pPV [3] on a PstI fragment to create plasmids pEX2-*iglA*::*lacZ* and pEX2-*pigR*::*lacZ*, which were then used with wild-type LVS to create strains LVS *iglA*::*lacZ* and LVS *pigR*::*lacZ* by allelic exchange. Strains LVS Δ*mglA pigR*::*lacZ* and LVS Δ*relA* Δ*spoT pigR*::*lacZ* were made using plasmid pEX2-*pigR*::*lacZ* together with strains LVS Δ*mglA* and LVS Δ*relA* Δ*spoT*.

### Transposon mutagenesis

One microgram of the *mariner* transposon delivery plasmid pSD [62] was used to transform the reporter strain LVS *iglA*::*lacZ*, and the resulting kanamycin-resistant isolates were patched onto CHAH supplemented with 100 µg/mL X-gal (Qiagen); approximately 9,000 mutants were screened for β-galactosidase activity. Chromosomal DNA was purified from isolates with decreased β-galactosidase activity relative to the non-mutagenized reporter strain, and the location of the transposon was determined by sequencing the chromosomal DNA as described previously [63].

### TAP

TAP was performed as described previously [20].

### Bacterial bridge two-hybrid assays

Cells were grown with aeration at 37°C in LB supplemented with carbenicillin (100 µg/mL), tetracycline (10 µg/mL), spectinomycin (100 µg/mL), and IPTG at the concentration indicated. Cells were permeabilized with SDS-CHCl_3_ and assayed for β-galactosidase activity as described previously [20]. Assays were performed at least three times in duplicate on separate occasions. Representative data sets are shown. Values are averages based on one experiment; duplicate measurements differed by less than 10%.

### Protein crosslinking and co-purification assays

Plasmid pF3 is a derivative of plasmid pF that confers resistance to nourseothricin. pF3 was constructed by first amplifying the nourseothricin resistance gene from plasmid pJK795 (provided by Julia Kohler) by the PCR and then cloning the corresponding PstI-SphI-digested PCR product into PstI-SphI-digested plasmid pF. Plasmid pF3-PigR-V directs the synthesis of the PigR protein with a VSV-G epitope-tag fused to its C-terminus, and was constructed by amplifying *pigR* from pF-PigR by the PCR using an oligo encoding the VSV-G tag and then inserting the corresponding BamHI-XmaI-digested PCR product into BamHI-XmaI-digested pF3. For the experiments described in Figure 9, plasmids pF3-PigR-V and pF3 were transformed into the indicated strains. Cells were grown to mid-log phase and proteins were crosslinked in cells from 3 mL of culture using 1% formaldehyde for 30 minutes followed by quenching for 15 minutes with 250 mM glycine. Cells were washed three times with PBS, sonicated extensively, and TAP was performed on the crosslinked proteins essentially as described earlier [20]. Following TAP, proteins were resuspended in NuPAGE LDS sample buffer (Invitrogen), incubated at 100° for 30 minutes to reverse the crosslinks, and subjected to SDS-PAGE and immunoblotting.

### *Francisella* β-galactosidase assays

Cells were grown to mid-log phase, permeabilized with SDS-CHCl_3_, and assayed for β-galactosidase activity essentially as described above for the bacterial two-hybrid assays.

### Immunoblots

Cell lysates and eluted proteins were separated by SDS-PAGE on 10% or 4%–12% Bis-Tris NuPAGE gels in MES or MOPS running buffer (Invitrogen) and transferred to nitrocellulose using the iBlot dry blotting system or the XCell II Blot Module (Invitrogen). Membranes were blocked with 25 mL of SuperBlock Blocking Buffer (Pierce) in TBS with 250 µL Surfact-Amps 20 (Pierce). Membranes were then probed with monoclonal antibodies against the VSV-G-tag (Sigma), the TAP-tag (using PAP; Sigma), the CBP portion of the TAP-tag (Open Biosystems), or Tul4 (provided by Karl Klose). Proteins were detected using goat polyclonal anti-rabbit IgG conjugated with horseradish peroxidase (Pierce), and visualized using SuperSignal West Pico or Femto Chemiluminescent Substrate (Pierce).

### RNA isolation and quantitative RT-PCR

RNA isolation and qRT-PCR were performed as described previously [20].

### Microarray experiments

Cells were grown to mid-log phase and RNA was isolated using Qiagen RNeasy Mini columns. RNA was purified from three separate cultures for each strain and DNase-treated using RQ1 DNase (Promega). cDNA was synthesized and prepared for hybridization as described previously [20]. Labeled probes in hybridization buffer (5× SSC, 50% formamide, 0.1% SDS, 0.6 µg/µL Salmon Sperm DNA) were applied to oligonucleotide arrays (provided by the NIAID Pathogen Functional Genomics Resource Center) using a Tecan HS400 Hybridization Station. Arrays were scanned using a GenePix 4000B microarray scanner (Molecular Devices) and data were analyzed with GeneSpring GX.

### Macrophage replication assays

J774 murine macrophage were seeded in DMEM (Mediatech) supplemented with 10% fetal bovine serum (Invitrogen) [DMEM-F] in a 96-well plate at a density of ∼10^5^ macrophages per well and incubated at 37° with 5% CO_2_ overnight. Macrophage were then infected with mutant strains or wild-type LVS (in triplicate; three wells per strain) at a multiplicity of infection of ∼15 and incubated at 37° with 5% CO_2_ for two hours. Macrophage were washed twice with PBS (Pierce) and then covered with DMEM-F containing gentamicin at 10 µg/mL. After 24 hours of incubation at 37° with 5% CO_2_, intracellular bacteria were enumerated by lysing the macrophage in PBS containing 1% saponin (Calbiochem) for five minutes at room temperature, diluting the lysate in PBS, and then plating onto CHAH.

### Mice challenge studies

Cultures were grown to early log phase (an OD_620_ of 0.2) harvested, and resuspended to the appropriate concentration in sterile PBS (Gibco). Inoculums were verified by direct plating on CHAH plates. Male BALB/cByJ mice (6–8 weeks old; Jackson Laboratory, Bar Harbor, ME) were caged in a microisolator in a pathogen-free environment in the animal facility at Harvard Medical School. Mice received the appropriate bacterial inoculum in sterile PBS via intradermal injection into the midbelly. Survival was monitored for 21 days after challenge.

### ppGpp assays

Intracellular levels of ppGpp were determined essentially as described [64]. Strains were grown with aeration at 37°C in modified Mueller-Hinton broth to an OD_600_∼0.2. Cells from 1 mL of culture were then pelleted and re-suspended in 250 µl MOPS-MGS [65] containing 55 mM mannose in place of mannitol, and incubated with aeration at 37°C for one hour in the presence of 25 µCi ^32^P KH_2_PO_4_ (Perkin Elmer). Nucleotides were then extracted by lysis of cells in 1 M formic acid and were resolved on a PEI cellulose thin layer chromatography plate (J.T. Baker) using 1.5 M KH_2_PO_4_ (pH 3.4) running buffer. Images were acquired using a GE Storm Phosphoimager.

## Supporting Information

Figure S1 Complementation of the effects of the Δ*caiC*, Δ*trmE*, Δ*cphA*, and Δ*pigR* mutations on virulence gene expression in *F. tularensis*. Quantitative RT-PCR analysis of *iglA* transcript abundance in wild-type (WT), Δ*trmE*, Δ*caiC*, Δ*cphA*, and Δ*pigR* mutant cells containing the indicated plasmids. Plasmid pF2-TrmE directs the synthesis of TrmE and plasmid pF2 serves as an empty vector control. Plasmids pCaiC and pCphA direct the synthesis of CaiC and CphA, respectively, and plasmid pFNLTP6 serves as an empty vector control. Plasmid pF-PigR directs the synthesis of PigR and plasmid pF serves as an empty vector control. Transcripts were normalized to those of *tul4*.
(0.85 MB EPS)

Figure S2 Complementation of the effects of the Δ*pigR* mutation by PigR-V. Quantification of *lacZ* expression in strain LVS *pigR::lacZ* containing the indicated plasmids. Plasmids pF-PigR and pF-PigR-V direct the synthesis of PigR and PigR-V, respectively. Plasmid pF served as the empty control vector.
(0.75 MB EPS)

Table S1 Microarray analysis of genes whose expression changes by a factor of 2.5 or more with a p-value <0.05 in either a Δ*mglA*, Δ*relA* Δ*spoT*, Δ*pigR*, Δ*caiC*, Δ*trmE*, or Δ*cphA* mutant background compared to wild-type. Negative values indicate genes that are positively regulated by MglA, ppGpp, PigR, CaiC, TrmE, or CphA, whereas positive values indicate genes that are negatively regulated. LVS ORFs are referred to by the LVS (FTL number) and Schu S4 (FTT number) locus tags for convenience, and gene names are included when available. “a” indicates those genes that belong to the MglA/SspA regulon [20]; “b” indicates that the p-value is between 0.05 and 0.1; and “c” indicates that the p-value is greater than 0.1. For all other fold changes the p-value is <0.05.
(0.06 MB DOC)
