# Supplementary material for: Small Molecule Control of Virulence Gene Expression in Francisella tularensis
Source: PLoS Pathog. 2009 Oct 30;5(10):e1000641. doi: 10.1371/journal.ppat.1000641 (PMC2763202; doi:10.1371/journal.ppat.1000641)
Supplement: Table S1 — Microarray analysis of genes whose expression changes by a factor of 2.5 or more with a p-value <0.05 in either a ΔmglA, ΔrelA ΔspoT, ΔpigR, ΔcaiC, ΔtrmE, or ΔcphA mutant background compared to wild-type. Negative values indicate genes that are positively regulated by MglA, ppGpp, PigR, CaiC, TrmE, or CphA, whereas positive values indicate genes that are negatively regulated. LVS ORFs are referred to by the LVS (FTL number) and Schu S4 (FTT number) locus tags for convenience, and gene names are included when available. “a” indicates those genes that belong to the MglA/SspA regulon [20]; “b” indicates that the p-value is between 0.05 and 0.1; and “c” indicates that the p-value is greater than 0.1. For all other fold changes the p-value is <0.05. (0.06 MB DOC) [file ppat.1000641.s003.doc]

| FTL Locus | FTT Locus | Gene name | Fold change in ∆*mglA* | Fold change in ∆*relA* ∆*spoT* | Fold change in ∆*pigR* | Fold change in ∆*caiC* | Fold change in ∆*trmE* | Fold change in ∆*cphA* |
| --- | --- | --- | --- | --- | --- | --- | --- | --- |
| FTL_0111^a^ | FTT1359 | *iglA* | -14.1 | -11.1 | -16.9 | -5.2 | -4.2 | -3.6 |
| FTL_0208 | FTT0297 |  | -3.7 | -5.1 | -3.9 | -3.9 | -2.6 | -2.6 |
| FTL_0118^a^ | FTT1352 |  | -2.9 | -4.5 | -4.0 | -2.8 | -2.4 | -4.0 |
| FTL_0449^a^ | FTT0383 | *pigR* | -3.4^b^ | -3.6 | -8.9^b^ | -2.3 | -2.3 | -2.3 |
| FTL_0113^a^ | FTT1357 | *iglC* | -3.8 | -3.5 | -3.8 | -3.0 | -3.7 | -1.5^c^ |
| FTL_0114^a^ | FTT1356 | *iglD* | -2.7 | -3.3 | -3.3 | -2.5 | -2.6 | -2.6 |
| FTL_0112^a^ | FTT1358 | *iglB* | -4.0^b^ | -3.0 | -4.6 | -2.1 | -1.8 | -1.3^c^ |
| FTL_0207^a^ | FTT0296 | *pcp* | -3.5^b^ | -2.9 | -4.0 | -3.2 | -2.1 | -1.7 |
| FTL_1361 | FTT0751c | *cspA* | -3.6 | -2.8 | -2.3 | -1.4 | -1.5 | -2.6 |
| FTL_1219^a^ | FTT0980 |  | -3.6 | -2.7 | -4.5 | -3.7 | -3.8 | -3.0 |
| FTL_1218^a^ | FTT0981 |  | -2.2 | -2.7 | -1.4 | -2.0 | -2.3 | -1.6 |
| FTL_0126 | FTT1344 | *pdpA* | -3.0 | -2.5 | -5.1 | -2.7 | -2.2 | -2.0 |
| FTL_1509 | not annotated |  | -2.0 | -2.3 | -2.8 | -2.3 | -1.8^b^ | -1.2^b^ |
| FTL_0129^a^ | FTT0252 | *leuA* | -1.6^c^ | -1.6 | -1.9 | -2.6 | -2.0 | -2.1 |
| FTL_0675^a^ | FTT1388 |  | -2.2 | -1.5 | -1.6 | -2.6 | -1.8 | -1.2^c^ |
| FTL_0131^a^ | FTT0251 | *ilvE* | -1.5 | -1.5 | -2.2 | -2.7 | -2.3 | -1.8 |
| FTL_0674^a^ | FTT1389 | *panB* | -1.7 | -1.4 | -1.5^b^ | -2.1 | -2.6 | -1.4^c^ |
| FTL_1579 | FTT0484 |  | -2.7 | -1.4 | -1.1 | +1.3 | -1.0^c^ | -1.0^c^ |
| FTL_0831 | FTT1130c | cphA | -1.6^b^ | -1.3^b^ | 1.0^c^ | +1.4 | +1.1 | -5.7 |
| FTL_0859 | FTT0595c | rubA | -2.8 | -1.3^b^ | +1.1^b^ | -1.4^b^ | -1.4 | -1.1^c^ |
| FTL_1539 | FTT0697 | ftsI | -1.4^c^ | -1.1^c^ | -1.6^c^ | -2.8 | -1.4^c^ | -2.0 |
